# The Association Between Early Sport Specialization and Injury and Career Outcomes Among National Football League Athletes

**DOI:** 10.1002/ejsc.70120

**Published:** 2026-01-12

**Authors:** Gnaneswar Chundi, Abhiram Dawar, Zachary Fuller, Tuckerman Jones, Shriyaus Lingam, Balazs Galdi

**Affiliations:** ^1^ Department of Orthopaedics Rutgers New Jersey Medical School Newark New Jersey USA

**Keywords:** athletic development, career longevity, injury risk, NFL, orthopedic outcomes, sports specialization

## Abstract

Early sport specialization during adolescence has been linked to overuse injuries in several sports, yet its long‐term impact in American football remains underexplored. The purpose of this study is to examine whether early specialization in football during high school is associated with higher injury rates and shorter careers than multi‐sport participation. This is a retrospective cohort study with level of evidence 3. We analyzed all NFL players drafted from 2011 to 2023 (*n* = 2556) who played ≥ 16 career games. Athletes were classified as multisport or single‐sport based on high school varsity participation using public records. Injury data were obtained from validated online databases. The primary outcome was injury incidence, measured as injuries per 1000 snaps (defined as individual plays participated in). Secondary outcomes included career length, total games played, and weighted career approximate value (AV). Injury rates were compared with position‐stratified incidence rate ratios (IRRs). Multisport athletes (63.6%) sustained fewer total injuries (IRR, 0.80, 95% CI, 0.76–0.85, and *p* < 0.001) and major injuries (IRR, 0.77, 95% CI, 0.71–0.82, and *p* < 0.001) compared to single‐sport athletes. Multisport athletes also played 12.2 more NFL games (95% CI, 9.2–15.1, *p* < 0.001, and *d* = 0.32) and had 0.7 additional career years (95% CI, 0.5–0.9, *p* < 0.01 and *d* = 0.28). NFL players who participated in multiple sports during high school had significantly lower injury rates and greater career durability. These findings support the body of evidence discouraging early sport specialization.

## Highlights

- NFL athletes who participated in multiple sports during high school sustained significantly fewer total and major injuries per 1000 snaps than single‐sport athletes.
- Multisport athletes demonstrated greater career durability, including more games played, longer careers, higher snap counts, and greater weighted career approximate values.
- Injury and durability advantages of multisport participation were most pronounced in speed‐ and agility‐dependent positions, including running backs, wide receivers, defensive backs, and linebackers.
- These findings support youth sport diversification and suggest that early football specialization is not required for elite performance or career longevity.

## 1 Background

Early sport specialization has become increasingly prevalent in youth athletics (Feeley et al. 2016; Swindell et al. 2019; Hayano et al. 2020). Many young athletes train year‐round in one sport, often at the expense of multisport participation, in pursuit of scholarships and elite performance in the sport of their choice (Feeley et al. 2016; Hayano et al. 2020; Brooks et al. 2018; Wojtys 2013; Jayanthi et al. 2019). This trend has raised concern among pediatric and sports medicine experts, as early specialization has been linked to risks such as overuse injuries and burnout in sports such as baseball and basketball (Feeley et al. 2016; Swindell et al. 2019; Hayano et al. 2020; Jayanthi et al. 2019; Rugg et al. 2018; Brenner 2016; Doveri and Neuffer 2018; Anderson et al. 2020). Brenner et al., in an American Academy of Pediatrics report, cautioned that youth who specialize too early may suffer physical and psychological detriments, and they recommended delaying single‐sport focus until late adolescence (Brenner 2016). Feeley and colleagues' review similarly noted that there is limited evidence of any competitive advantage to early single‐sport training, but emerging data suggest that it may increase injury risk (Feeley et al. 2016).

In contrast, a growing body of literature indicates that a multisport background during youth could be beneficial for long‐term athletic development (McLellan et al. 2022; Güllich et al. 2022). Güllich et al. reported in a meta‐analysis that professional athletes tended to engage in multiple sports during childhood and started specializing later than their national‐level peers. These athletes accumulated a broader foundation of motor skills. Such findings align with the “sampling” theory of athlete development, which posits that early diversification can enhance injury resilience and reduce monotonic training stress (Güllich et al. 2022).

Epidemiologic studies across sports have begun to examine early specialization outcomes in elite populations (Swindell et al. 2019; Rugg et al. 2018; McLellan et al. 2022; McDonald et al. 2019; Moseid et al. 2019). In baseball, for example, a survey of professional players found that those who played multiple sports in high school went on to play significantly more total games in Major League Baseball (mean 362.8 vs. 300.8) than those who specialized in baseball alone (McLellan et al. 2022). The multisport baseball players also had a lower prevalence of overuse upper extremity injuries (50% vs. 63%, *p* < 0.01). Similarly, among National Basketball Association athletes, Rugg et al. 2018 observed that first‐round draftees with multisport backgrounds in high school had fewer major injuries and greater career longevity than those who specialized early (Rugg et al. 2018). These studies suggest that the benefits of multisport participation may translate into fewer injuries and longer careers at the professional level (Rugg et al. 2018; McLellan et al. 2022; Confino et al. 2019; Sang et al. 2025).

Evidence in American football, however, remains limited and somewhat mixed. Football is a high‐impact sport with a high rate of injury in professional athletics, including a substantial risk of season‐ending injuries (Bullock et al. 2021). It has been hypothesized that athletes who only played football through their youth might be more prone to such injuries due to repetitive stress or lack of cross‐training adaptation, but definitive data are sparse. One study by Stein et al. focused on NFL first‐round draft picks (2008–2017) and found that an overwhelming majority (88%) had been multisport athletes in high school. Interestingly, the authors reported no significant differences between multisport and single‐sport first rounders in terms of NFL games missed due to injury, total games played, or career length. However, the analysis was restricted to first‐round picks, a subset of players with fewer single‐sport individuals and generally longer careers by virtue of talent and opportunity (Steinl et al. 2021). Athletes who specialize in football at an early age may possess a heightened awareness of techniques and playing styles that help mitigate injury risk, likely stemming from the greater volume of training they have undergone. However, limiting the analysis to first‐round draft picks may not fully capture this effect, as it excludes a broader range of players whose training histories and injury patterns could provide a more comprehensive understanding. Thus, it remains unclear whether early sport specialization has a noticeable effect on injury rates and career outcomes across the overall NFL population, regardless of draft rounds.

Therefore, the purpose of this study was to evaluate the association between early sport specialization and two key outcomes in NFL players: injury incidence and career durability. A comprehensive cohort of NFL athletes drafted from 2011 onward were analyzed to test the hypothesis that early specialization is associated with higher injury rates and shorter careers in professional football.

## 2 Methods

### 2.1 Study Design and Cohort Selection

We conducted a retrospective cohort study of NFL players drafted from 2011 to 2023, initially including all 3590 athletes selected during this period. Players were excluded if they participated in fewer than 16 career games, equivalent to one full NFL regular season, or if they played exclusively as kickers or punters, positions that do not reflect the typical physical demands and roles of other positions on the field. After applying these criteria, 2556 athletes remained eligible. Only drafted players starting in 2011 were included, as their higher online visibility increased the likelihood of comprehensive documentation of their high school athletic backgrounds. All player data were de‐identified. Institutional review board approval was not required due to the publicly available nature of the dataset.

### 2.2 Early Specialization Classification

Players were classified as either “multi‐sport” or “single‐sport” athletes based on their documented high school sports participation. Early sport specialization was defined as participation exclusively in football throughout U.S. high school (grades 9–12, typically aged 14–18 years), without involvement in other varsity‐level sports. Athletes who competed in at least one additional varsity sport besides football were classified as multisport athletes.

For high school sport participation, reviewers used a standardized search protocol across MaxPreps, Athletic.net, and Hudl. Each athlete's full name was entered directly into the platform's search bar, and potential matches were verified using graduation year, high school name, and geographic location to ensure correct player identification. When multiple athlete profiles existed or information was incomplete, the same confirmation process was applied. If ambiguity remained, reviewers conducted a uniform supplemental internet search (e.g., “<player name> high school sports,” local news articles, and school athletic pages) to confirm sport participation. Only sports verified through this approach were included in the final classification, and disagreements were resolved by consensus.

Because publicly available records predominantly document high school participation, we used a binary classification system based on participation during late adolescence and were unable to distinguish specialization that occurred before high school (< 14 years of age). As such, our classification aligns with what prior studies describe as later specialization. Earlier specialization patterns—particularly those influenced by sex‐specific differences in timing of growth and puberty—could not be assessed in this dataset.

### 2.3 Position Classification

Player positions were obtained from publicly available NFL draft data and classified into eight standard positional groups: defensive back (DB), defensive line (DL), linebacker (LB), offensive line (OL), quarterback (QB), running back (RB), tight end (TE), and wide receiver (WR). Safeties, cornerbacks, and nickelback defenders were all included in the DB category. Defensive tackles and defensive ends were grouped as DL, and centers, guards, and offensive tackles were grouped as OL. This approach mirrors published methodology in NFL injury surveillance studies and ensures adequate sample sizes within each category.

### 2.4 Injury Data and Definitions

Injury data for each athlete were systematically collected from professional sports databases, primarily ESPN.com and prosportstransactions.com, the latter of which has previously been validated in the literature as an accurate database of player injuries (Bullock et al. 2021). Reviewers searched p ro s ports t ransactions.com and ESPN.com using each player's name, recording all documented football‐related injuries that resulted in missed time or placement on injured reserve. To maintain consistency, the same extraction order and definitions were applied across all athletes. Any discrepancies across sources were cross‐checked and resolved through consensus.

Two injury‐related outcomes were analyzed in this study. The first outcome, total injuries, encompassed all officially documented football‐related injuries sustained by players during their NFL careers, including minor injuries (those resulting in fewer than four consecutive games missed, such as a three‐game ankle sprain) and more significant injuries (severe enough to result in placement on injured reserve (IR) or to cause at least four consecutive missed games). Each injury incident was counted individually, even if it recurred across different seasons. Injuries related to illness (including COVID‐19), concussions, upper extremity fractures, jaw fractures, and facial bone fractures were excluded, as these injuries do not typically reflect chronic issues associated with sport specialization.

### 2.5 Career Durability and Performance Metrics

We assessed multiple indicators of career durability and achievement. Total games played reflected overall availability, including both starts and substitutions. Career length was defined as the number of NFL seasons from a player's first to most recent active year, rounded to the nearest full year (e.g., 2011–2015 = 5 years), with players active through 2024–2025 counted accordingly.

Weighted career approximate value (AV), obtained from Pro‐Football‐Reference.com, was used to evaluate overall productivity and performance. AV is a proprietary composite statistic generated by Pro Football Reference that assigns each player a single value for each season based on position‐specific playing time, box score statistics, and team offensive or defensive performance. Weighted career AV is calculated by Pro Football Reference as the sum of a player's season AV values with greater weight applied to their highest value (“peak”) seasons. AV and weighted career AV were taken directly from Pro Football Reference and were not recalculated by the authors. Although not a primary end point, weighted career AV offered context on whether early specialization influenced career success.

Total snaps—the cumulative number of official NFL plays a player participates in (each “snap” represents a single offensive, defensive, or special teams’ play)—were used to quantify workload and calculate injury rates. All metrics were sourced from Pro‐Football‐Reference.com.

### 2.6 Outcome Measures

The primary outcome was injury incidence, compared between multisport and single‐sport athlete groups. Injury incidence was quantified as the rate of injuries per unit of play. For each player, the injury rate was calculated as (total injuries ÷ total snaps) × 1,000, yielding injuries per 1000 snaps played. Similarly, the major injury rate was computed as (major injuries ÷ total snaps) × 1000. These rates standardized injury counts to individual playing time, accounting for variability in career length and on‐field exposure. Higher injury rates reflected a greater propensity for injury per play. This method aligns with prior American football epidemiology research that reports injury incidence per play or per 1000 plays as a measure of exposure (McCunn et al. 2017; Jadischke et al. 2020; Whelan et al. 2023; Mack et al. 2018).

### 2.7 Validation of Multisport Classification

To evaluate the accuracy of high school sport participation classification, two independent reviewers performed external validation on a random sample of 100 athletes classified as single‐sport athletes. Using a random number generator, 100 players were selected from the full dataset. For each player, reviewers conducted structured internet searches across high school athlete databases, archived newspaper coverage, school athletic websites, and MaxPreps profiles to identify evidence of participation in additional varsity‐level sports. Discrepancies were resolved by consensus. Out of 100 sampled athletes, 97 were confirmed as single‐sport athletes based on publicly available records, yielding a 97% confirmation rate. These findings support fidelity of the classification schema.

### 2.8 Statistical Analysis

The primary analyses compared (1) injury incidence (total and major injuries per 1000 snaps) and (2) career durability metrics (career length, total games played, and total snaps) between multisport and single‐sport athletes.

The primary analysis evaluating differences in injury incidence between multisport and single‐sport athletes was conducted using Poisson regression. The injury count was modeled as the dependent variable, with the sport specialization group (multisport vs. single‐sport) as the independent variable. Total NFL snaps played was included as an offset term, allowing injury rates to be adjusted for exposure time. Models were not additionally adjusted for age or other player characteristics, as age at draft was similar between groups and the primary objective was to estimate crude rate differences by specialization history. Model outputs are presented as incidence rate ratios (IRRs) with corresponding 95% confidence intervals (CIs) and *p* values. This approach provides an appropriate rate comparison accounting for differing levels of play among athletes.

Continuous variables, including total games played, career length, weighted career approximate value (AV), and injury rates (total and major injuries per 1000 snaps), were calculated as mean ± standard deviation (SD). Group comparisons for continuous variables were performed using independent two‐sample *t*‐tests. To account for unequal sample sizes and variances between groups, Welch's *t*‐test was employed. For each comparison, the mean difference between groups and the corresponding 95% confidence interval (CI) was reported, as this provides the most interpretable estimate of the magnitude of differences between groups. Given the presence of nonnormal distributions and potential outliers, particularly for injury rates, games played, and career length, nonparametric Mann–Whitney U tests were performed as sensitivity analyses for all continuous outcomes listed above. Categorical variables, such as the distribution of multisport versus single‐sport athletes across position groups, were compared using chi‐square tests.

Secondary analyses included position‐stratified comparisons using the methods described above. To contextualize all position‐stratified analyses, we applied false discovery rate (FDR) correction using the Benjamini–Hochberg method. Adjusted *p*‐values are provided in Supporting Information S1: Table 1. All statistical tests were two‐tailed, with a significance threshold set at *p* < 0.05. Analyses were conducted using Python 3.12.3 (SciPy 1.14.1 and statsmodels 0.14.4 libraries).

## 3 Results

### 3.1 Cohort Characteristics

Among 2556 NFL draftees from 2011 to 2023, 64% were multisport athletes. When stratified by position, the proportion varied significantly (Table 1). Among multisport athletes, the most common secondary sports played in high school included track and field (67%), basketball (58%), and baseball (12%) (Table 2).

**TABLE 1:** Difference between single‐sport and multisport cohorts by NFL position.

Position	Total, n	Multisport, *n* (%)	Single‐sport, *n* (%)	*p*‐value
DB	535	356 (67%)	179 (33%)	< 0.01
DL	462	292 (63%)	170 (37%)	0.03
LB	334	195 (58%)	139 (42%)	0.04
OL	435	252 (58%)	183 (42%)	0.31
QB	79	57 (72%)	22 (28%)	0.08
RB	234	140 (60%)	94 (40%)	0.37
TE	157	117 (75%)	40 (25%)	< 0.01
WR	320	226 (71%)	94 (29%)	< 0.01
All positions	2556	1626 (64%)	930 (36%)	< 0.01

**TABLE 2:** Distribution of sports played in high school among multisport athletes.

Sport played	*n*	% of multisport athletes
Track & field	1088	67%
Basketball	947	58%
Baseball	193	12%
Wrestling	52	3%
Soccer	21	1%
Lacrosse	18	1%
Volleyball	12	1%
Other	100	6%

### 3.2 Incidence Rate Ratios

Overall, multisport athletes had a significantly lower incidence rate for total injuries (IRR 0.80, 95% CI 0.76–0.85, and *p* < 0.001) and major injuries (IRR 0.77, 95% CI 0.71–0.82, and *p* < 0.001).

Stratified by position, the association of multisport participation with a lower injury rate remained evident in several groups. Among defensive backs, multisport athletes had significantly lower total injury rates (IRR, 0.81; 95% CI, 0.71–0.92; and *p* = 0.001) and major injury rates (IRR, 0.68; 95% CI, 0.58–0.79; and *p* < 0.001). Defensive linemen showed similar differences in both total (IRR, 0.81; 95% CI, 0.71–0.94; and *p* = 0.005) and major injury rates (IRR, 0.80; 95% CI, 0.67–0.95; and *p* = 0.010). Among linebackers, multisport athletes had a 26.7% lower total injury rate (IRR, 0.73; 95% CI, 0.63–0.85; and *p* < 0.001) and a 32.8% lower major injury rate (IRR, 0.67; 95% CI, 0.56–0.80; and *p* < 0.001). For running backs, differences were even more pronounced between the total injury rate (IRR, 0.66; 95% CI, 0.55–0.79; and *p* < 0.001) and the major injury rate (IRR, 0.62; 95% CI, 0.50–0.77; and *p* < 0.001). Among wide receivers, multisport athletes experienced lower total injury rates (IRR, 0.68; 95% CI, 0.58–0.81; and *p* < 0.001) and major injury rates (IRR, 0.67; 95% CI, 0.55–0.81; and *p* < 0.001).

In contrast, no significant differences were observed among offensive linemen (total injury IRR, 0.84; 95% CI, 0.73–0.96; *p* = 0.011; major injury IRR, 0.86; 95% CI, 0.73–1.01; and *p* = 0.063), quarterbacks (total injury IRR, 0.99; 95% CI, 0.66–1.47; *p* = 0.946; major injury IRR, 1.55; 95% CI, 0.92–2.62; and *p* = 0.101), or tight ends (total injury IRR, 1.02; 95% CI, 0.80–1.30; *p* = 0.901; major injury IRR, 1.05; 95% CI, 0.78–1.42; *p* = 0.743) (Table 3, 4).

**TABLE 3:** Total injury rates (per 1000 snaps) and incidence rate ratios by position.

Position	Multisport rate	Single‐sport rate	IRR (95% CI)	*p*‐value
All players	1.11	1.57	0.80 (0.76–0.85)	< 0.01
DB	0.99	1.36	0.81 (0.71–0.92)	< 0.01
DL	1.09	1.37	0.81 (0.71–0.94)	0.01
LB	1.04	1.50	0.73 (0.63–0.85)	< 0.01
OL	1.06	1.55	0.84 (0.73–0.96)	0.01
QB	0.74	0.69	0.99 (0.66–1.47)	0.95
RB	1.45	2.27	0.66 (0.55–0.79)	< 0.01
TE	1.47	1.49	1.02 (0.80–1.30)	0.90
WR	1.18	1.96	0.68 (0.58–0.81)	< 0.01

**TABLE 4:** Major injury rates (per 1000 snaps) and incidence rate ratios by position.

Position	Multisport rate	Single‐sport rate	IRR (95% CI)	*p*‐value
All players	0.85	1.24	0.77 (0.71–0.82)	< 0.01
DB	0.72	1.17	0.68 (0.58–0.79)	< 0.01
DL	0.85	1.08	0.80 (0.67–0.95)	0.01
LB	0.76	1.13	0.67 (0.56–0.80)	< 0.01
OL	0.88	1.24	0.86 (0.73–1.01)	0.06
QB	0.60	0.39	1.55 (0.92–2.62)	0.10
RB	1.03	1.76	0.62 (0.50–0.77)	< 0.01
TE	1.12	1.06	1.05 (0.78–1.42)	0.74
WR	0.92	1.55	0.67 (0.55–0.81)	< 0.01

To contextualize the position‐specific findings, we compared raw *p*‐values with false discovery rate (FDR)–adjusted *p*‐values (*q*‐values) across all subgroup analyses. The overall cohort comparisons were not subjected to FDR correction, consistent with their designation as primary analyses. Among position‐stratified comparisons, all statistically significant associations in defensive backs, defensive linemen, linebackers, running backs, and wide receivers remained significant after FDR adjustment (*q* < 0.01 for all). In contrast, the nonsignificant findings for offensive linemen, quarterbacks, and tight ends also remained nonsignificant after adjustment (all *q* > 0.05). (Supporting Information S1: Table 1a.)

### 3.3 Career Durability

Across the full cohort, multisport athletes demonstrated significantly greater career durability than single‐sport athletes. Multisport athletes played 12.2 more NFL games (95% CI, 9.2–15.1; *p* < 0.01; and *d* = 0.32), had 0.7 more career years (95% CI, 0.5–0.9; *p* < 0.01; and *d* = 0.28), accumulated 649 additional snaps (95% CI, 467–830; *p* < 0.01; and *d* = 0.28), and achieved 4.7 higher weighted career AV (95% CI, 3.3–6.2; *p* < 0.01; and *d* = 0.25) compared with single‐sport athletes (Tables 5, 6, 7, 8).

**TABLE 5:** Difference in career games played between multisport and single‐sport athletes.

Position	Mean difference (Games)	95% CI	*p*‐value	Effect size (d)
All players	+12.2	9.2–15.1	< 0.01	0.32
DB	+17.4	11.4–23.3	< 0.01	0.49
DL	+16.1	8.6–23.7	< 0.01	0.40
LB	+14.0	6.1–22.0	< 0.01	0.38
OL	+7.8	0.3–15.3	0.04	0.20
QB	+5.7	−17.6–29.0	0.62	0.13
RB	+14.7	6.3–23.1	< 0.01	0.45
TE	−1.6	−15.5–12.3	0.82	−0.04
WR	+11.3	3.1–19.5	< 0.01	0.30

**TABLE 6:** Difference in NFL career length between multisport and single‐sport athletes.

Position	Mean difference (Years)	95% CI	*p*‐value	Effect size (d)
All players	+0.7	0.5–0.9	< 0.01	0.28
DB	+1.0	0.6–1.4	< 0.01	0.41
DL	+1.2	0.6–1.7	< 0.01	0.42
LB	+1.0	0.4–1.5	< 0.01	0.39
OL	+0.3	−0.2–0.9	0.20	0.13
QB	+0.1	−1.8–2.0	0.93	0.02
RB	+0.7	0.1–1.3	0.02	0.30
TE	−0.1	−1.1–0.9	0.89	−0.03
WR	+0.6	0.0–1.2	0.04	0.22

**TABLE 7:** Difference in total NFL snaps between multisport and single‐sport athletes.

Position	Mean difference (snaps)	95% CI	*p*‐value	Effect size (d)
All players	+649	467–830	< 0.01	0.28
DB	+995	607–1383	< 0.01	0.43
DL	+836	461–1211	< 0.01	0.41
LB	+757	264–1250	< 0.01	0.33
OL	+507	−37–1050	0.07	0.18
QB	+396	−1116–1907	0.60	0.14
RB	+524	196–852	< 0.01	0.41
TE	−63	−728–602	0.85	−0.03
WR	+609	160–1058	< 0.01	0.29

**TABLE 8:** Difference in weighted career approximate value (AV) between multisport and single‐sport athletes.

Position	Mean difference (AV)	95% CI	*p*‐value	Effect size (d)
All players	+4.7	3.3–6.2	< 0.01	0.25
DB	+5.4	3.0–7.7	< 0.01	0.37
DL	+7.2	3.7–10.7	< 0.01	0.37
LB	+6.2	1.9–10.6	< 0.01	0.30
OL	+4.1	0.5–7.7	0.03	0.21
QB	+4.2	−13.4–21.8	0.63	0.13
RB	+5.9	1.8–10.0	< 0.01	0.36
TE	+0.6	−3.5–4.8	0.76	0.05
WR	+3.7	−0.4–7.9	0.08	0.20

When stratified by the position group, the largest differences favoring multisport athletes were observed among defensive backs, defensive linemen, linebackers, and running backs. These groups showed consistent advantages across all durability metrics, including games played, career length, total snaps, and weighted AV, with effect sizes ranging from small to moderate (*d* = 0.30–0.49). Wide receivers demonstrated similar trends, with significant differences in games played, snaps, and career length.

In contrast, quarterbacks and tight ends showed no statistically significant differences between multisport and single‐sport athletes across any durability metric (Tables 5, 6, 7, 8). These findings mirror the injury rate results, in which no consistent advantage was observed for multisport athletes in these two positions. The absence of significant differences among quarterbacks and tight ends may reflect limited statistical power, as these position groups had smaller sample sizes than others in the cohort.

To contextualize the position‐stratified durability findings, we compared raw *p*‐values with FDR‐adjusted *p*‐values (*q*‐values) across all subgroup analyses (Supporting Information S1: Table 1b). For defensive backs, defensive linemen, linebackers, and running backs, all four durability metrics (total games, career length, total snaps, and weighted AV) remained statistically significant after FDR adjustment (all *q* < 0.05). Among wide receivers, differences in total games and total snaps remained significant (*q* = 0.012 and *q* = 0.013, respectively), whereas differences in career length and weighted AV did not (*q* = 0.075 and *q* = 0.105). For offensive linemen, only the difference in weighted AV remained significant after adjustment (*q* = 0.041), while differences in games played, career length, and snaps were no longer statistically significant (*q* = 0.055, 0.260, and 0.090, respectively). Quarterbacks and tight ends showed no statistically significant durability differences after FDR adjustment across any metric.

## 4 Discussion

### 4.1 Injury and Career Outcomes

In this retrospective cohort of NFL draftees (2011–2023), athletes who participated in multiple sports during high school demonstrated significantly lower rates of orthopedic injuries and increased career longevity in the NFL compared with those who specialized in football alone. Overall injury incidence was lower among multisport athletes (incurring approximately 20% fewer injuries per play, *p* < 0.001). Similarly, multisport players sustained fewer major injuries requiring extended recovery. These benefits correlated with improved durability: on average, multisport athletes played more NFL games and had increased career longevity than their single‐sport peers. The hypothesized “protective effect” of a multisport background was especially pronounced at positions characterized by high running demands and open‐field play (i.e., RB, WR, DB, and LB). After applying FDR‐adjusted *p*‐values, the durability and injury rate advantages for defensive backs, defensive linemen, linebackers, wide receivers, and running backs remained statistically significant, whereas findings for quarterbacks, offensive linemen, and tight ends did not retain statistical significance. These position‐specific findings highlight that though the overall trend favors multisport participation, the trend can vary by roles. Collectively, the data support that multisport participation during adolescence is associated with reduced injury risk and greater career durability in professional football players without compromising elite achievement.

Overall, the findings support the consensus that early sport diversification enhances physical development and reduces injury risk. One recent systematic review of elite athletes by McLellan et al. found that delaying specialization is consistently associated with a reduced risk of injuries. All eight studies in the review, which examined injury outcomes, reported lower injury rates among athletes who specialized later or participated in multiple sports (McLellan et al. 2022). Similarly, another meta‐analysis of youth athletes (ages 7–18) by Carder et al. concluded that sport “samplers” had a significantly lower risk of injury than single‐sport specializers. The implication is that engaging in varied sports during development can distribute repetitive load across different muscle groups and movements, mitigating overuse injuries seen with year‐round single‐sport training (Carder et al. 2020).

Several studies done on professional athletes in different leagues in literature echo this sentiment. For instance, in a 2019 study by on MLB players, athletes who were single‐sport specialists experienced increased rates of chronic injuries such as stress reactions and tendinitis in the shoulder and the elbow. By contrast, multisport athletes may alternate stresses on the body, allowing tissue recovery and balanced muscular development (Confino et al. 2019). Similarly, Rugg et al. (2018) examined NBA first‐round draftees and reported that the multisport NBA athletes participated in a greater percentage of games and were about half as likely to suffer a major injury during their careers, and a significantly higher proportion were still active in the league after several years (Rugg et al. 2018). These findings corroborate the findings of the current work, namely, an improved reduced injury rate and increased career longevity at the professional level in those who play multiple sports. Furthermore, although research in youth populations differs from our NFL cohort, a biomechanics study by DiStefano et al. demonstrated that multisport youth were more likely to develop proper landing mechanics than single‐sport peers—a foundational neuromuscular trait linked to lower long‐term risk of ACL injuries and lower extremity overuse pathology. Although professional athletes were not studied directly, this work supports a plausible developmental pathway through which early sport diversification may contribute to improved musculoskeletal resilience later in an athlete's career (DiStefano et al. 2018).

On the other hand, several studies suggest that the advantages seen in this study's cohort may be diminished in certain contexts. For instance, Steinl et al. examined a similar question in a cohort of NFL first‐round draft picks (2008–2017) and reported no significant difference in injury history and professional career length between those who were multisport athletes in high school and those who specialized in football (Steinl et al. 2021). Focusing only on top draft picks—who may have longer careers due to talent and opportunity—could obscure the impact of specialization. By using all drafted players, this study more clearly analyzes multisport participation.

Likewise, in professional baseball and soccer, survey‐based analyses have found no clear disparities in career longevity or success metrics between early specializers and those who sampled multiple sports (Confino et al. 2019; Knapik et al. 2020). One survey of Major League Baseball players noted no difference in the number of seasons played at the major league level between athletes who specialized early and those who did not. Approximately half of the studies on elite athletes' career longevity have reported no association between youth sport specialization and the length of an athlete's career (McLellan et al. 2022).

Two separate studies found that athletes who specialized earlier had better performance in sports like elite marathon running and soccer (Knapik et al. 2020; Noble and Chapman 2018). Similarly, in other sports with early peak ages or where technical skill development at a young age is critical (e.g., women's gymnastics, figure skating, or ballet), early specialization is often considered necessary for reaching the highest levels (LaPrade et al. 2016). These cases suggest that the optimal path to elite performance in terms of specialization may vary by sport, a sentiment echoed in recent commentary by Lundberg et al. who highlighted that athlete development is shaped by sport‐specific physical, psychological, and contextual demands and that early specialization is neither inherently beneficial nor harmful across all sports but must be interpreted within each sport's developmental framework (Lundberg et al. 2025).

These psychological and developmental mechanisms may also help explain differences in career longevity observed in our cohort. Early single‐sport specialization often introduces a range of stressors and constraints that can adversely affect young athletes' mental health and overall development (Doveri and Neuffer 2018; LaPrade et al. 2016; Daley et al. 2023; McFadden et al. 2015; Gould 2010). Specializing youths may face increased pressure to perform, repetitive year‐round training, and high expectations from coaches and parents, all of which contribute to elevated stress and risk of burnout (Hayano et al. 2020; Doveri and Neuffer 2018; Gould 2010). Studies have noted that early sport specialization leads to social isolation, increased anxiety, sleep disturbances, and decreased family time, which can erode an athlete's well‐being and motivation (Daley et al. 2023; Brenner et al. 2019; Watson and Brickson 2019). Epidemiologic data confirm that rates of burnout and early sports retirement are higher among youth who specialize earlier than those who participate in multiple sports (Barynina and Vaitsekhovskii 1992; Wall and Côté 2007). Although psychological outcomes were not a primary aim of this study, the existing literature suggests that these factors may offer an alternative insight into why multisport athletes in our cohort demonstrated longer NFL careers and greater overall durability.

Collectively, these findings highlight that the relationship between specialization and elite performance is nuanced, sport‐dependent, and shaped by biological, psychological, and environmental factors rather than any single training trajectory.

### 4.2 Position‐Specific Interpretation of Effects

American football is unique in that each position comes with different physical builds and demands. As such, the lack of a measurable difference in multisport quarterbacks, offensive linemen, and tight ends warrants further discussion. These positions have unique physical demands and injury profiles that may explain the attenuated influence of early sport history. Quarterbacks in the NFL generally cover less total distance at high speeds during games and are subject to fewer high‐frequency impacts than positions like running back or defensive back. Their injury risk often stems from acute, high‐impact events (such as being sacked) or from specialized, repetitive motions (throwing mechanics) focused on the upper extremity (Radel et al. 2020; Kirsch et al. 2018). It is plausible that the diverse movement training obtained through multiple sports has less carryover to quarterback‐specific injury mechanisms. A multisport background might improve general athleticism in quarterbacks, but it may not significantly protect against the types of injuries quarterbacks typically sustain, such as contact‐driven trauma or throwing‐related arm injuries (Radel et al. 2020; Kirsch et al. 2018; Kelly et al. 2004). This could help explain why multisport quarterbacks in our cohort did not show a lower injury rate than single‐sport quarterbacks (IRR ∼0.99, *p* = 0.946, and *q* = 0.950).

For offensive linemen and tight ends, the physical demands of football are characterized by repetitive blocking, trench contact, and large static and dynamic loads. These players operate in constrained space with less requirement for the kind of open‐field agility or varied movement patterns seen in skill positions. Their injuries are frequently due to collision forces (e.g., piled tackles and cut blocks) and the cumulative effects of high‐mass impacts on joints (Karton et al. 2018; Ackerman et al. 2023). It is conceivable that no amount of early agility training or cross‐sport neuromuscular conditioning can fully mitigate the intrinsic injury risks of line play (Ackerman et al. 2023). For instance, an offensive lineman's ankle may be equally vulnerable to another player falling on it, regardless of whether that lineman also ran track in high school. Moreover, due to positional demands, linemen who specialize in football might inadvertently undergo multidisciplinary training (focused on weight training and line drills) that confer some of the same benefits as playing a different sport (Fullagar et al. 2017). This could narrow the observable difference between multisport and single‐sport linemen. Another consideration is that these positions often involve athletes at the extremes of body size, which itself is a risk factor for certain injuries (such as joint degeneration) that could overshadow the influence of early training background (Lambach et al. 2015; Gómez et al. 1998). Ultimately, this observed position‐specific variation underscores that the association of early specialization with injury can depend on the athletic demands and injury mechanisms inherent to each position.

In contrast to the abovementioned positions, our injury data demonstrated lower injury rates in those who had multisport participation in speed‐ and agility‐dominant roles, including defensive backs, linebackers, defensive linemen, wide receivers, and running backs. These groups showed significantly lower total and major injury rates than single‐sport athletes, and these differences remained statistically significant after FDR adjustment (*q* < 0.01 for all positions). These findings suggest multisport training may have the greatest association with lower injury rate in positions where neuromuscular control, change of direction, proprioception, and deceleration mechanics are central to injury prevention. Conversely, positions dominated by collision‐driven or size‐dependent injury mechanisms may be inherently less associated with early movement diversification.

Together, these positional trends reinforce that the benefits of multisport participation, though robust overall, manifest differently across the varied biomechanical demands of NFL roles.

### 4.3 Clinical Implications

From a public health and player development standpoint, these findings carry important implications. They provide empirical support at the highest competitive level for recommendations long advocated by pediatric and sports medicine experts: early sport specialization should be avoided in favor of diverse athletic participation through adolescence (Brenner 2016). The American Academy of Pediatrics, American Orthopaedic Society for Sports Medicine, and other authorities have cautioned that focusing on a single sport year‐round before late adolescence raises injury risk and can undermine long‐term athletic success (Jayanthi et al. 2019). Our study's outcome that multisport athletes not only reach the NFL at high rates but also enjoy more durable careers directly reinforces this guidance. It suggests that young athletes do not need to specialize early to achieve elite performance; on the contrary, those who broadened their athletic experiences tended to fare better in terms of staying healthy and productive once at the professional level.

This evidence can empower clinicians, coaches, and parents to encourage skill development through multiple sports, emphasizing fundamental motor skills, strength, and coordination over sport‐specific mastery in the prepubertal and adolescent years. By reducing monotonous stress on any one anatomical region, a multisport approach lowers cumulative trauma and allows for more complete physical development. The current findings, therefore, lend weight to existing policies that advocate for multisport participation and place limits on year‐round training in a single sport for young athletes (Jayanthi et al. 2019; Brenner 2016). Ultimately, a cultural shift toward valuing all‐around athletic development in youth could help reduce injury rates at all levels of sport and produce healthier, more adaptable athletes. Sports organizations might consider frameworks that incentivize multisport involvement or at least structured rest periods and cross‐training in the off‐season, especially for sports like football where the seasonal nature permits playing a different sport during off seasons.

Practical implementation of such frameworks could include sport‐specific limits on year‐round training, mandated off‐season rest periods, and structured windows that allow or encourage participation in a second sport during developmental years. Youth sport governing bodies may also adopt evidence‐informed guidelines similar to those proposed by Lundberg et al., emphasizing delayed selection, flexible training pathways, and reduced early talent triage. Additionally, schools and clubs can collaborate to minimize scheduling conflicts between sports, offer integrated athletic development programs, and provide coaching education centered on longer term athlete development rather than early specialization (Lundberg et al. 2025).Collectively, these strategies can create an ecosystem that supports multisport engagement across a wide range of youth and adolescent sports.

### 4.4 Study Limitations

Despite the strengths of a large sample and novel focus on NFL outcomes, this study has several limitations. First, the retrospective design precludes definitive conclusions about causality. Although associations between multisport participation and improved outcomes are evident, it cannot be proven that early specialization caused injuries or shorter careers. It is possible that inherent differences in athletes (e.g., overall athleticism or coaching quality) influenced both their likelihood to specialize and their durability in the NFL.

Second, all data were derived from public records and media sources. There may be misclassification or reporting bias in determining high school sport histories, and some athletes' secondary sports might have been underreported. Furthermore, injury reporting in the NFL is not perfectly standardized. We focused on injuries leading to missed games or IR designation, but minor injuries managed “in‐play” would not be captured, potentially underestimating total injury incidence.

Third, the cohort consisted exclusively of NFL‐drafted players, an elite subset of athletes. These results may not generalize to all youth football players or to those who do not reach professional status. Additionally, because the cohort consists exclusively of male professional athletes, the findings may not generalize to female athletes or to sports with different sex‐specific injury profiles. Well‐established sex differences in biomechanics, neuromuscular control, and injury mechanisms may influence how early specialization affects long‐term outcomes, and therefore, comparisons with studies including mixed‐sex or female cohorts should be interpreted cautiously.

Another important limitation concerns the heterogeneity in how prior studies define “early specialization.” Definitions vary substantially across the literature, ranging from exclusive single‐sport participation before the age 12 to year‐round training in one sport and to the absence of multi‐sport participation during adolescence. Because our study classified specialization solely based on high school sport participation, some comparisons with earlier studies, particularly those using younger age thresholds, may not reflect equivalent constructs. This definitional inconsistency complicates direct comparison of findings across studies and highlights the need for standardized, consensus‐based criteria when evaluating the associations of sport specialization.

Furthermore, though career length and games played are practical metrics of durability, they can be influenced by factors like coaching decisions, team needs, or noninjury‐related reasons (e.g., personal choice or off‐field issues) that we could not fully account for.

Finally, the binary distinction between single‐sport and multisport participation does not capture the multidimensional nature of specialization described in recent consensus frameworks. Contemporary models emphasize that specialization encompasses not only sport participation patterns but also physical load, motivational factors, training volume, psychosocial environment, and parental/coach influence. Our approach, constrained by publicly available data, could not incorporate these components. As highlighted by Kliethermes et al. 2025 more comprehensive specialization indices, integrating physical, motivational, and social dimensions, may better characterize the developmental context and could alter or refine associations observed in future studies (Kliethermes et al. 2025). In summary, the observational nature of this study and possible confounders warrant a careful interpretation of the associations noted, without overextending to causal claims.

## 5 Conclusion

In summary, this study provides strong evidence that NFL players who avoided early single‐sport specialization and had lower injury rates and longer careers than those who specialized in football during high school. These findings, in concert with prior research, underscore the potential long‐term health and performance benefits of a multisport youth background. Although causation cannot be definitively established from this retrospective analysis, its results align with biomechanical and developmental theory and lend support to existing recommendations against early sport specialization. By highlighting improved durability in multisport athletes at the highest level of football, this study contributes meaningful data to the ongoing dialog on youth sports training strategies, reinforcing the message that broad‐based athletic development is a prudent path toward both sporting success and injury prevention. Future research should prioritize longitudinal studies tracking athletes over time to clarify how early specialization affects long‐term outcomes, while incorporating biomechanical, physiological, and imaging assessments to explore underlying mechanisms. Expanding this work to include female athletes and other sports with different specialization timelines is also essential.

## Author Contributions

G.C. and A.D. conceived the study and conducted data analysis. G.C., A.D., and S.L., led manuscript development. All authors contributed to writing the original draft, reviewing the manuscript, and approving the final version for submission. T.J., Z.F., and B.G. also provided supervision and critical guidance throughout the study.

## Funding

The authors have nothing to report.

## Ethics Statement

The authors have nothing to report.

## Consent

The authors have nothing to report.

## Conflicts of Interest

The authors declare no conflicts of interest.

## Permission to Reproduce Material From Other Sources

The authors have nothing to report.

## Clinical Relevance

This study provides evidence that early multisport participation is associated with fewer injuries in elite footballers.

## Supporting information


Supporting Information S1

## Data Availability

All data used in this study are publicly available.
