# Supplementary material for: The Association Between Early Sport Specialization and Injury and Career Outcomes Among National Football League Athletes
Source: Eur J Sport Sci. 2026 Jan 12;26(2):e70120. doi: 10.1002/ejsc.70120 (PMC12795778; doi:10.1002/ejsc.70120)
Supplement: Supplementary file 1 — Supporting Information S1 [file EJSC-26-e70120-s001.docx]

Supplemental Table 1a. Injury Incidence (Poisson Regression, Multi-sport vs Single-sport)

Effect estimate is incidence rate ratio (IRR) for multi-sport vs single-sport athletes. Primary overall analyses are not FDR-adjusted; FDR adjustment applies only to position-stratified comparisons.

| **Outcome** | **Position** | **Effect estimate (IRR, 95% CI)** | **Raw p-value** | **FDR-adjusted p (q-value)** |
| --- | --- | --- | --- | --- |
| **Total injuries** | Overall* | 0.80 (0.76–0.85) | <0.001 | – |
|  | DB | 0.81 (0.71–0.92) | 0.002 | 0.003 |
|  | DL | 0.81 (0.71–0.94) | 0.005 | 0.008 |
|  | LB | 0.73 (0.63–0.85) | <0.001 | <0.001 |
|  | OL | 0.84 (0.73–0.96) | 0.011 | 0.015 |
|  | QB | 0.99 (0.66–1.47) | 0.946 | 0.950 |
|  | RB | 0.66 (0.55–0.79) | <0.001 | <0.001 |
|  | TE | 1.02 (0.80–1.30) | 0.901 | 0.946 |
|  | WR | 0.68 (0.58–0.81) | <0.001 | <0.001 |
| **Major injuries** | Overall* | 0.77 (0.71–0.82) | <0.001 | – |
|  | DB | 0.68 (0.58–0.79) | <0.001 | <0.001 |
|  | DL | 0.80 (0.67–0.95) | 0.010 | 0.016 |
|  | LB | 0.67 (0.56–0.80) | <0.001 | <0.001 |
|  | OL | 0.86 (0.73–1.01) | 0.064 | 0.085 |
|  | QB | 1.55 (0.92–2.62) | 0.101 | 0.115 |
|  | RB | 0.62 (0.50–0.77) | <0.001 | <0.001 |
|  | TE | 1.05 (0.78–1.42) | 0.743 | 0.743 |
|  | WR | 0.67 (0.55–0.81) | <0.001 | <0.001 |

Supplemental Table 1b. Career Durability Outcomes (Linear Comparisons, Multi-sport – Single-sport)

Effect estimates are mean differences (multi-sport minus single-sport) with 95% CIs and Cohen’s d. Primary overall comparisons are not FDR-adjusted; FDR applies only to position-stratified analyses.

| **Outcome** | **Position** | **Effect estimate (mean difference, 95% CI; d)** | **Raw p-value** | **FDR-adjusted p (q-value)** |
| --- | --- | --- | --- | --- |
| **Total games** | Overall* | +12.2 games (9.2–15.1; d = 0.32) | <0.001 | – |
|  | DB | +17.4 games (11.4–23.3; d = 0.49) | <0.001 | <0.001 |
|  | DL | +16.1 games (8.6–23.7; d = 0.40) | <0.001 | <0.001 |
|  | LB | +14.0 games (6.1–22.0; d = 0.38) | <0.001 | 0.001 |
|  | OL | +7.8 games (0.3–15.3; d = 0.20) | 0.042 | 0.055 |
|  | QB | +5.7 games (–17.6–29.0; d = 0.13) | 0.624 | 0.713 |
|  | RB | +14.7 games (6.3–23.1; d = 0.45) | <0.001 | 0.001 |
|  | TE | –1.6 games (–15.5–12.3; d = –0.04) | 0.819 | 0.819 |
|  | WR | +11.3 games (3.1–19.5; d = 0.30) | 0.007 | 0.012 |
| **Career length** | Overall* | +0.7 years (0.5–0.9; d = 0.28) | <0.001 | – |
|  | DB | +1.0 years (0.6–1.4; d = 0.41) | <0.001 | <0.001 |
|  | DL | +1.2 years (0.6–1.7; d = 0.42) | <0.001 | <0.001 |
|  | LB | +1.0 years (0.4–1.5; d = 0.39) | <0.001 | 0.001 |
|  | OL | +0.3 years (–0.2–0.9; d = 0.13) | 0.195 | 0.260 |
|  | QB | +0.1 years (–1.8–2.0; d = 0.02) | 0.929 | 0.929 |
|  | RB | +0.7 years (0.1–1.3; d = 0.30) | 0.026 | 0.048 |
|  | TE | –0.1 years (–1.1–0.9; d = –0.03) | 0.893 | 0.929 |
|  | WR | +0.6 years (0.0–1.2; d = 0.22) | 0.047 | 0.075 |
| **Total snaps** | Overall* | +649 snaps (467–830; d = 0.28) | <0.001 | – |
|  | DB | +995 snaps (607–1383; d = 0.43) | <0.001 | <0.001 |
|  | DL | +836 snaps (461–1211; d = 0.41) | <0.001 | <0.001 |
|  | LB | +757 snaps (264–1250; d = 0.33) | 0.003 | 0.005 |
|  | OL | +507 snaps (–37–1050; d = 0.18) | 0.068 | 0.090 |
|  | QB | +396 snaps (–1116–1907; d = 0.14) | 0.599 | 0.684 |
|  | RB | +524 snaps (196–852; d = 0.41) | 0.002 | 0.005 |
|  | TE | –63 snaps (–728–602; d = –0.03) | 0.851 | 0.851 |
|  | WR | +609 snaps (160–1058; d = 0.29) | 0.008 | 0.013 |
| **Weighted AV** | Overall* | +4.7 AV (3.3–6.2; d = 0.25) | <0.001 | – |
|  | DB | +5.4 AV (3.0–7.7; d = 0.37) | <0.001 | <0.001 |
|  | DL | +7.2 AV (3.7–10.7; d = 0.37) | <0.001 | <0.001 |
|  | LB | +6.2 AV (1.9–10.6; d = 0.30) | 0.005 | 0.010 |
|  | OL | +4.1 AV (0.5–7.7; d = 0.21) | 0.026 | 0.041 |
|  | QB | +4.2 AV (–13.4–21.8; d = 0.13) | 0.629 | 0.719 |
|  | RB | +5.9 AV (1.8–10.0; d = 0.36) | 0.005 | 0.010 |
|  | TE | +0.6 AV (–3.5–4.8; d = 0.05) | 0.761 | 0.761 |
|  | WR | +3.7 AV (–0.4–7.9; d = 0.20) | 0.079 | 0.105 |
